# Ectoparasitism and infections in the exoskeletons of large fossil cingulates

**DOI:** 10.1371/journal.pone.0205656

**Published:** 2018-10-18

**Authors:** Fábio Cunha Guimarães de Lima, Kleberson de Oliveira Porpino

**Affiliations:** 1 Programa de Pós-Graduação em Ecologia e Conservação, Universidade Federal Rural do Semi-Árido, Mossoró, Brazil; 2 Departamento de Ciências Biológicas, Universidade do Estado do Rio Grande do Norte, Mossoró, Brazil; Universite de Nantes, FRANCE

## Abstract

Studies on paleopathological alterations in fossil vertebrates, including damages caused by infections and ectoparasites, are important because they are potential sources of paleoecological information. Analyzing exoskeleton material (isolated osteoderms, carapace and caudal tube fragments) from fossil cingulates of the Brazilian Quaternary Megafauna, we identified damages that were attributed to attacks by fleas and dermic infections. The former were compatible with alterations produced by one species of flea of the genus *Tunga*, which generates well-delimited circular perforations with a patterned distribution along the carapace; the latter were attributable to pathogenic microorganisms, likely bacteria or fungi that removed the ornamentation of osteoderms and, in certain cases, generated craters or pittings. Certain bone alterations observed in this study represent the first record of flea attack and pitting in two species of large glyptodonts (*Panochthus* and *Glyptotherium*) and in a non-glyptodontid large cingulate (*Pachyarmatherium*) from the Quaternary of the Brazilian Intertropical Region. These new occurrences widen the geographic distribution of those diseases during the Cenozoic and provide more evidence for the co-evolutionary interaction between cingulates and parasites registered to date only for a small number of other extinct and extant species.

## Introduction

Among the representatives of the South American Pleistocene Megafauna, the cingulates stand out as one of the most diverse and peculiar clades. In this group, three main lineages are traditionally recognized: Dasypodids, likely a paraphyletic group [1], with fossil and recent species, and the extinct pampatheres and glyptodonts [2].

Paleopathological studies about cingulates, as for other taxa, can provide paleoecological and evolutionary insights concerning organism diseases from past to present. Cingulates show a complex exoskeleton overlaying their head, back and tail [3] formed by the fusion or articulation of dermal ossifications (osteoderms), whose external ornamentation varies considerably among species and are abundantly represented in the fossil record. However, few works in paleopathology have focused on exoskeleton diseases, although the inclusion of this structure in pathological analyses might offer evidence of other types of diseases not preserved in endoskeleton bones (e.g., dermal diseases). In fact, some authors already report cutaneous pathologies affecting the exoskeleton of cingulates, chiefly glyptodonts, including: fractures and dermic lesions in a carapace of *Panochthus tuberculatus* [4], formation of crests and orifices generating an irregular aspect on the surface of osteoderms in *Neosclerocalyptu*s [5] and *Glyptodon* cf. *G*. *clavipes* [6], three types of dermal lesions in a carapace of *Panochthus* [7], and cases of ectoparasitism by fleas, which generated bioerosions in osteoderms of the dorsal carapace in late Miocene dasypodids [8] and probably in *Holmesina*, a Pleistocene pampathere [9]. Moreover, some additional reports found flea infections in armadillo osteoderms in an archaeological context [10, 11]

This work reports on the first cases of parasitism by fleas and other cutaneous lesions on exoskeleton elements of large fossil cingulates, including two glyptodonts (*Panochthus* and *Glyptotherium*) and a large cingulate of uncertain affinities (*Pachyarmatherium*), based on a survey of a great number of exoskeleton materials from Quaternary sites in northeastern Brazil. Based on this perspective, we provide new insights concerning on the paleoecology and evolution of this group.

## Materials and methods

### Study area, geological background and fossil material

**Fig 1:**
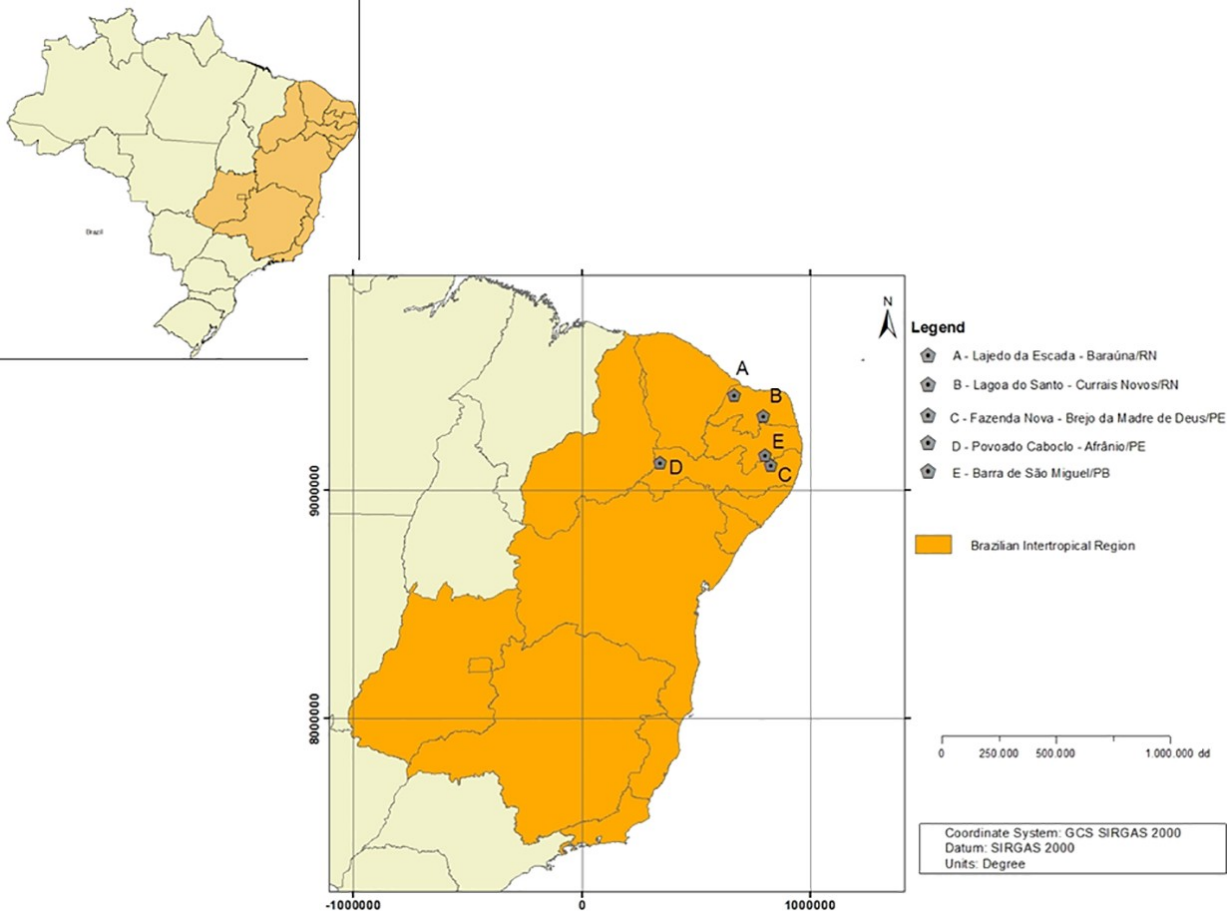
Location map of the collection sites within the Brazilian Intertropical Region (BIR).

The fossils analyzed herein are housed in several of the most representative paleontological collections of the Brazilian Intertropical Region (BIR) (*sensu* [12]; Fig 1), belonging to the Museu Câmara Cascudo (MCC), Natal City, Rio Grande do Norte State and Departamento de Geologia-UFPE (DEGEO-UFPE), Recife City, Pernambuco State. The fossils studied were assigned to the glyptodonts *Panochthus* sp. and *Glyptotherium* sp. and the large *incertae sedis* cingulate *Pachyarmatherium brasiliense* [13]. All fossils were collected in paleontological sites located in three states within the BIR (Fig 1). This region embraces an expansive area of 2,140,000 km^2^, with *Cerrado* and *Caatinga* ecosystems and is considered an important geographic area for paleontological research [12].

In the BIR, several types of fossil deposits occur [14, 15]. The fossils analyzed in this work were collected from sedimentary infillings of limestone caves and natural tanks and in alluvial deposits. Most available absolute dates place the megafauna remains from tank and cave deposits from the BIR in the Late Pleistocene [15, 16], with few exceptions assigned middle Pleistocene [17] and early Holocene ages [18, 19]. Therefore, we considered the material studied herein as belonging to the Late Pleistocene. The specimens included the following: isolated osteoderms and carapace fragments of *P*. *brasiliense* and *Glyptotherium* sp. from a limestone cave in the Lajedo da Escada (5°14´31”S, 37°44’20”W), Baraúna Municipality, Rio Grande do Norte State; isolated osteoderms of *P*. *brasiliense* collected in a tank in the Fazenda Nova locality (8°11’2”S, 36°10’01”W), Brejo da Madre de Deus Municipality, Pernambuco State; one partial carapace and isolated osteoderms of the genus *Panochthus* collected in a tank in the Lagoa do Santo locality (19°61’46”S, 44° 03’09” W), Currais Novos Municipality, Rio Grande do Norte State; two fragments from the lateral portion of a caudal tube of *Panochthus* collected in a fluvial deposit in Barra do São Miguel Municipality, Paraíba State; and isolated osteoderms belonging to this last genus collected in a muddy layer near a river [20] in the Povoado Caboclo locality (08°30’54”S, 41°00’18”W), Afrânio Municipality, Pernambuco State. Natural tanks are common fossiliferous deposits in the BIR and correspond to natural depressions caused by erosion of fractures in basement rocks by physical-chemical weathering, forming small pluvial water reservoirs [21, 22, 23]. These tanks are filled with clasts and bioclasts deposited primarily by hydraulic transport under a debris flow regime [23].

The exoskeleton elements of *Glyptotherium* collected in the Lajedo da Escada site were found in association with several postcranial bones that might belong to a single individual [24]. The partial carapace of *Panochthu*s in the Lagoa do Santo site were also found in association with isolated osteoderms and with several postcranial bones [25].

### Methods

We restricted our analyses to osteoderms with a preservation level of 50% or more, which allowed a reliable analysis of pathologies and more secure taxonomical identifications. We conducted a macroscopic inspection [26], useful to detect alterations in bone surfaces. Additionally, we also used a stereoscopic magnifying glass, a digital microscope Dino-Lite Basic with DinoCapture 2.0 Software and digital caliper with accuracy of 0.01 mm to scale out the lesions. We based our diagnoses on comparisons with previously reported cases in the literature [10, 8, 27]. We used taphonomically and pathologically unaltered osteoderms of *Panochthus* and *Glyptotherium* to compare with the affected ones. The unaltered osteoderm of *Panochthus* (132-V-UERN) was collected in a natural tank from Taperoá Municipality, Paraíba State, Brazil, and is deposited in the collection of the Laboratório de Paleontologia of Universidade do Estado do Rio Grande do Norte (LABPALEO-UERN), Mossoró city, Rio Grande do Norte State, Brazil. The osteoderm of *Glyptotherium* was collected in the Lajedo da Escada site, as was the remaining material of this genus analyzed here, and deposited in MCC.

**Fig 2:**
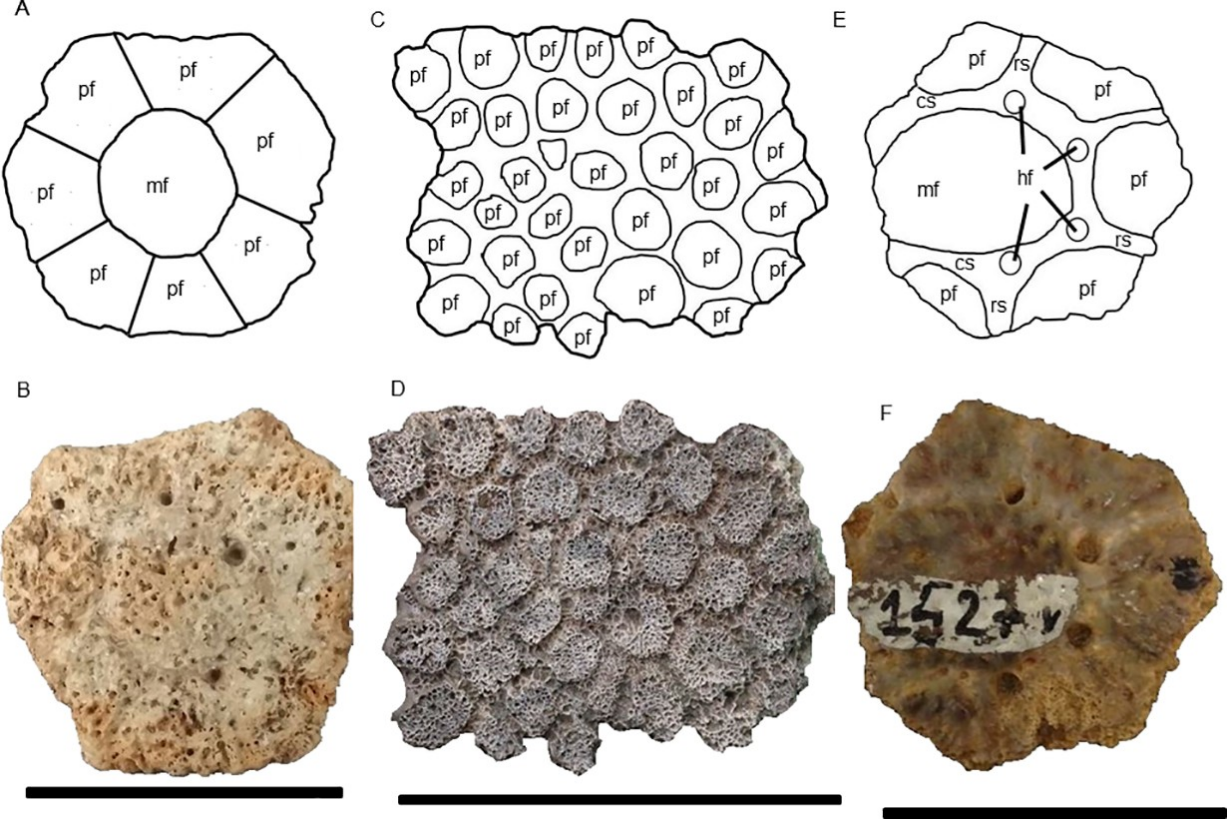
Schematic ilustration and terminologies for the external surface ornamentation of the osteoderms. (A) and (B), *Glyptotherium* sp. (C) and (D), *Panochthus* sp.; (E) and (F), *Pachyarmatherium brasiliense*. mf: main figure; pf: peripheral figure; cs: central sulcus; rs: radial sulcus; hf: hair follicle. Scale bars: A = 5 cm; B = 4 cm and C = 3 cm.

In this work, we adopted particular anatomical terminologies for different parts of the exoskeleton. For the carapace as a whole and caudal tube, we used the terminology proposed by Porpino et al. [28] (see Fig 2 of this work). For description of osteoderms (isolated or in carapace fragments), we adopted the anatomical terms commonly used in the literature [29, 14, 28, 30] regarding the ornamentation of their external surface (Fig 2). In this context, we used the term main figure for the largest figures in osteoderms instead of the term central figure. We considered as fragments of the carapace the elements comprising two or more fused osteoderms.

For the purposes of the present work, which concerned *ante mortem* alterations, we treated pitting and perforation as terms referring to different types of bone bioerosion. Pitting or cratering was cavities generated horizontally by erosions in the external cortical surface and with the potential to penetrate into trabecular bone (See Fig 3A in [27]). By contrast, a perforation is a vertical cavity that penetrated the bone that was also created by erosion, although showing well-delimited circular edges.

## Results

### Panochthus

Material analyzed–one partial carapace (MCC 1603-V), 162 isolated osteoderms, of which 53 show alterations (DEGEO-UFPE 2434; DEGEO-UFPE 5914; DEGEO-UFPE 7440; MCC 1385 V; MCC 1411-V; MCC 1412-V; MCC 1419-V; MCC 1422-V; MCC 1425-V; MCC 1427-V; MCC 1430-V; MCC 1575-V; MCC 1598-V; MCC 1602-V; MCC 1604-V; MCC 1606-V; MCC 1607-V; MCC 1609-V; MCC 1614-V; MCC 1615-V; MCC 1629-V; MCC 1631-V; MCC 1632-V; MCC 1634-V; MCC 1637-V; MCC 1638-V; MCC 1653-V; MCC 1654-V; MCC 1657-V; MCC 1659-V; MCC 1661-V; MCC 1665-V; MCC 1672-V; MCC 1674-V; MCC 1685-V; MCC 1686-V; MCC 2957-V; MCC 2967-V; MCC 3218-V and MCC 3222-V) plus 11 additional uncatalogued isolated specimens and two uncatalogued fragments of caudal tube deposited in DEGEO-UFPE.

Glyptodonts of the genus *Panochthus* have osteoderms that show a homogenous ornamentation along most of the carapace. The osteoderms next to lateral edge of the carapace have differentiated central and peripheral figures in contrast to the rest of lateral and dorsal regions, which, in most species, have only small figures [31, 28] likely homologous to the peripheral ones. We observed alterations in isolated osteoderms and in different points of the partial carapace MCC 1603-V, which in this case, varied according to the ornamentation.

#### Description of the lesions

Missing was most of the anterior region of the partial carapace MCC 1603-V. Consequently, we could not evaluate whether this section was pathologically affected. Among the preserved parts, the posterodorsal and posterolateral lateral regions were affected by pitting associated or not with loss of ornamentation and bone response in some points.

**Fig 3:**
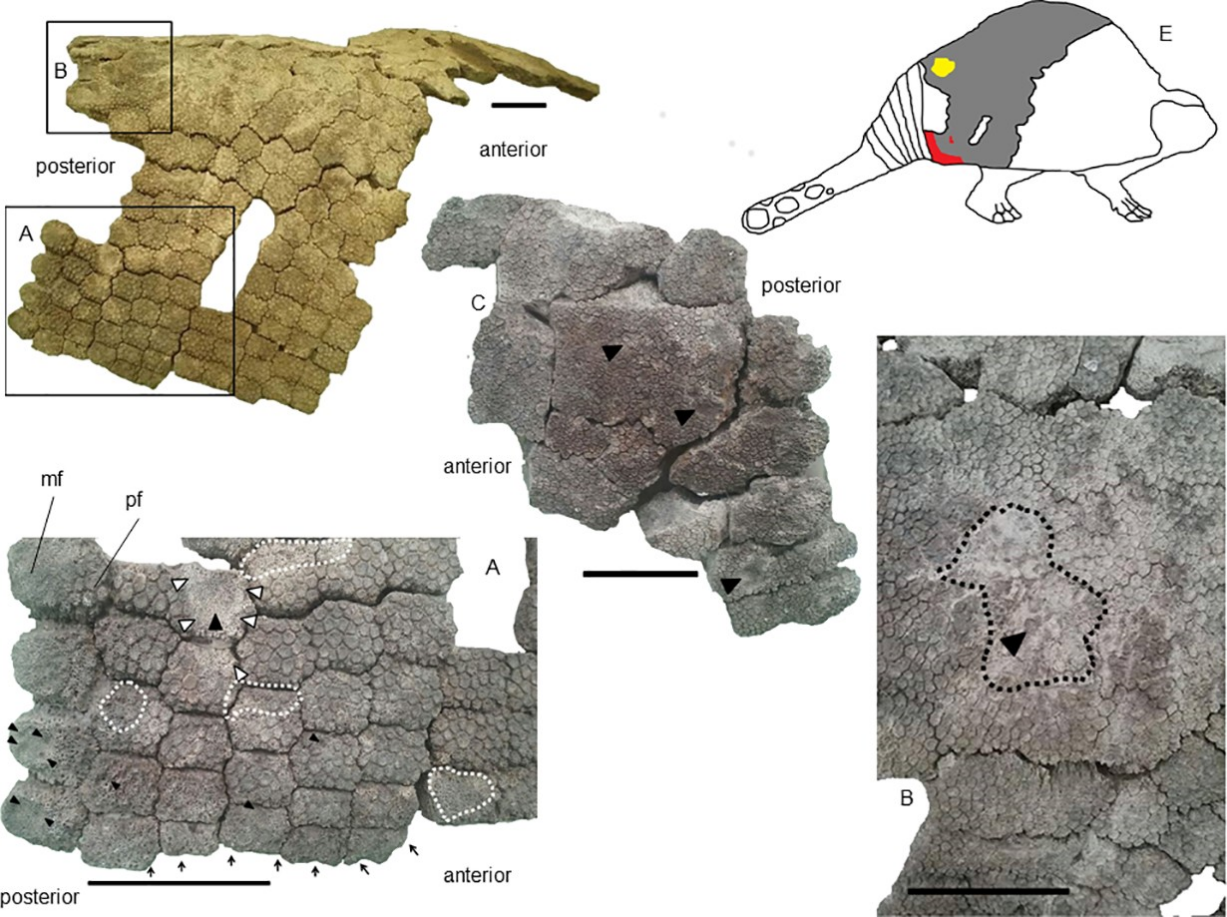
Lesions along the carapace of *Panochthus* sp. (MCC 1603-V). (A) Right posterolateral region. (B) posterodorsal region. (C) Left posterolateral region. The black arrowheads indicate micro/macrocavities generated by pitting. The white arrowheads indicate a smooth crest of bone response surrounding the pitting. The white dashed lines delimit the extension of the lesion by pitting or irregular wearing. The black dashed lines delimit the extension of the bone response over the carapace in the posterodorsal region. The black arrows point to the loss of ornamentation. (E) Schematic illustration of *Panochthus* (based on [32]) showing the area corresponding to the preserved carapace fragment MCC-1603-V; the color red indicates the region affected by pitting and the yellow color points the areas showing bone response. mf: main figure; pe: peripheral figure. Scale bars = 10 cm.

The right posterolateral and posterodorsal regions showed loss of the ornamentation, which conferred to the affected areas an irregular and rough aspect due to the exposition of the trabecular tissue. The osteoderms of the edge of right posterolateral region showed a high frequency of microcavities in the main figures. Additionally, on this region, we observed multilocated loss of ornamentation, primarily in the lateral edges. Next to the posterodorsal region, we verified a crater surrounded by a crest created by bone response (Fig 3A). In the left posterolateral region, the ornamentation showed few signs of erosion; depressions formed cavities, although they were less frequent and larger than the similar features on the right posterolateral region (Fig 3C). We considered these cavities as pitting.

We observed a modified area with approximately 10 cm extending from the posterodorsal to the right posterolateral region, showing bone response with a contorted aspect, suggesting calcium deposition (Fig 3B). The ornamentation around this new bone growth remained unaltered, with a low frequency of pits. Inside this area, pits were associated with exposure of trabecular bone and microfractures. By contrast, in the margins of this area, the frequency of pits was high, characterizing a multifocal distribution of pits.

All injured isolated osteoderms from Currais Novos and Brejo da Madre de Deus sites had the external surface eroded resulting in soft to severe exposure of trabecular bone by pitting or other erosive process.

**Fig 4:**
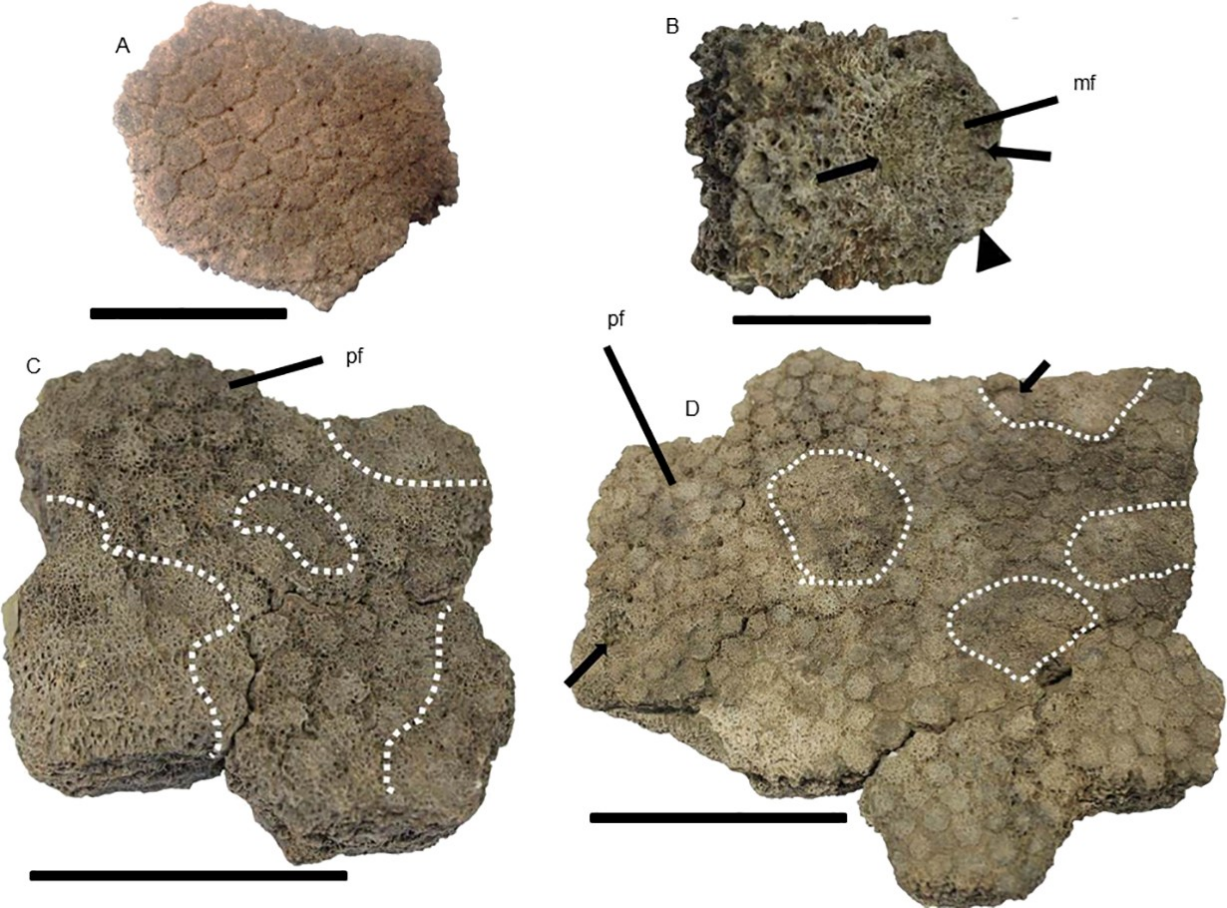
Osteoderms of *Panochthus* sp., external surface. (A) Unaltered osteoderm 132-V-UERN. (B) Pitting in the main figure of the osteoderm from the posterior edge MCC-1685-V; the arrows indicate two cavities separated by a crest pointed by the arrowhead (see text). (C) and (D), fragments from dorsal region of the carapace (MCC-1412-V and MCC 1412-V, respectively) with lesions delimited by the white dashed lines; the black arrows in D point orifices probably generated by pitting. pf: peripheral figure. Scale bars: A = 3 cm; B, C and D = 4 cm.

**Fig 5:**
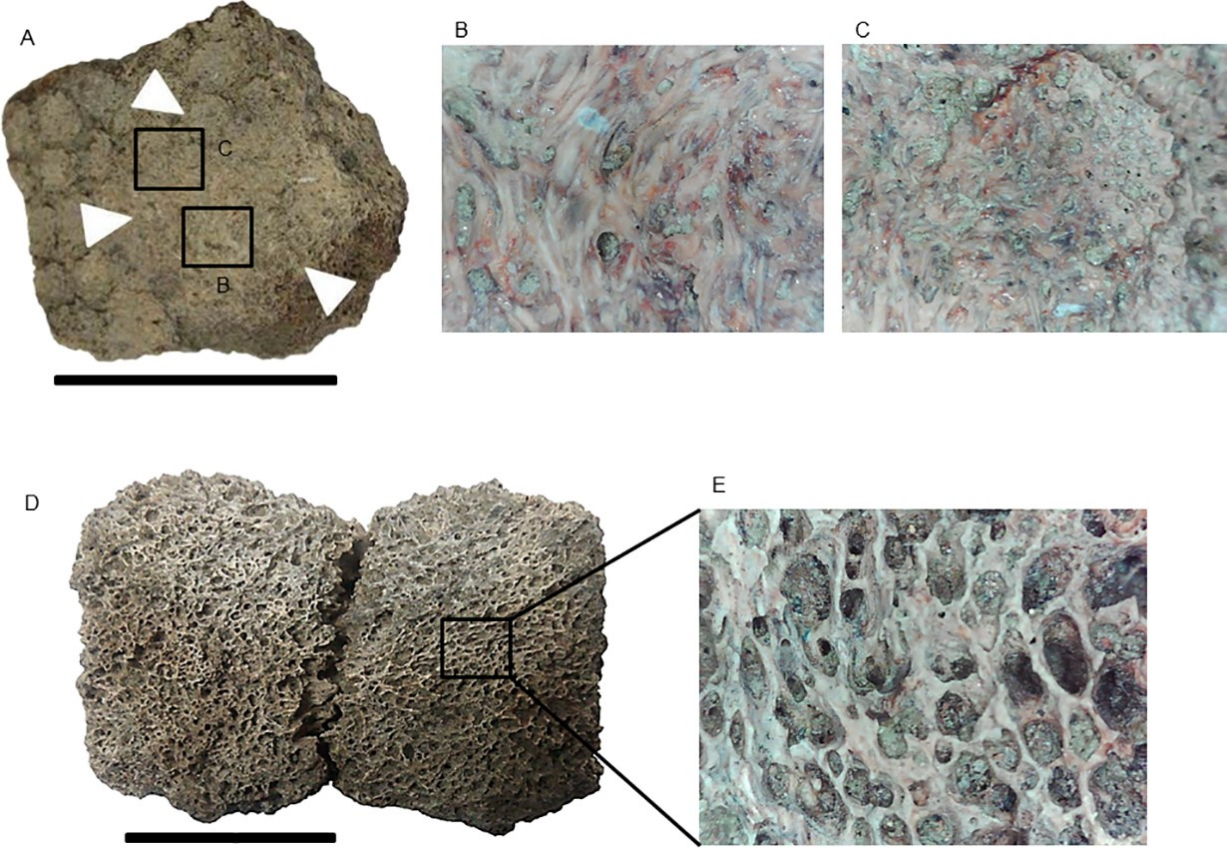
Osteoderms of *Panochthus* sp., external surface. (A) MCC 1653-V osteoderm with bone response indicated by the white arrowheads. (B) Digital microscopy image of MCC 1653-V showing the filaments of calcium deposition of a bone response (Zoom: 55x). (C) Digital microscopy image of MCC 1653-V showing a peripheral figure half unaltered and half with bone response. (Zoom: 35x). (D) Pathological exposure of spongy bone in MCC 1632-V. (E) Digital microscopy image of MCC 1632-V evidencing the porosity of the surface caused by erosion with no sign of bone reaction (Zoom: 55x). Scales bars: A = 3 cm; B = 2 cm.

Among the isolated osteoderms and carapace fragment from Currais Novos alterations led to partial loss of the ornamentation in wide extensions of their external surface and the formation of crests by irregular and differential erosion (MCC 1412-V; Fig 4C and MCC 1427-V; Fig 4D). Few isolated osteoderms from the dorsal region showed pitting. As for *Glyptotherium* (next section), in *Panochthus*, few osteoderms showed bone growth in response to the lesions, and those responses, when present, conferred to the affected surface a contorted aspect (MCC 1653-V; Fig 5A). We identified some cases in which the loss of the ornamentation exposed the spongy bone (MCC 1632-V; Fig 5D). In digital microscopical view, we observed a twisted and contorted pattern of filaments overlapping the spongy bone (Fig 5B and 5C). Moreover, the digital microscopy of the eroded area without bone response showed the typical porous aspect of the spongy bone, but without sign of bone response (Fig 5E). We also observed pitting in the main figure of posterior region osteoderms, which modified the normal aspect of the main figure by producing a depression with a midline crest (MCC 1653-V; Fig 4B); this feature was also observed in some osteoderms in the carapace MCC 603-V (Fig 3).

**Fig 6:**
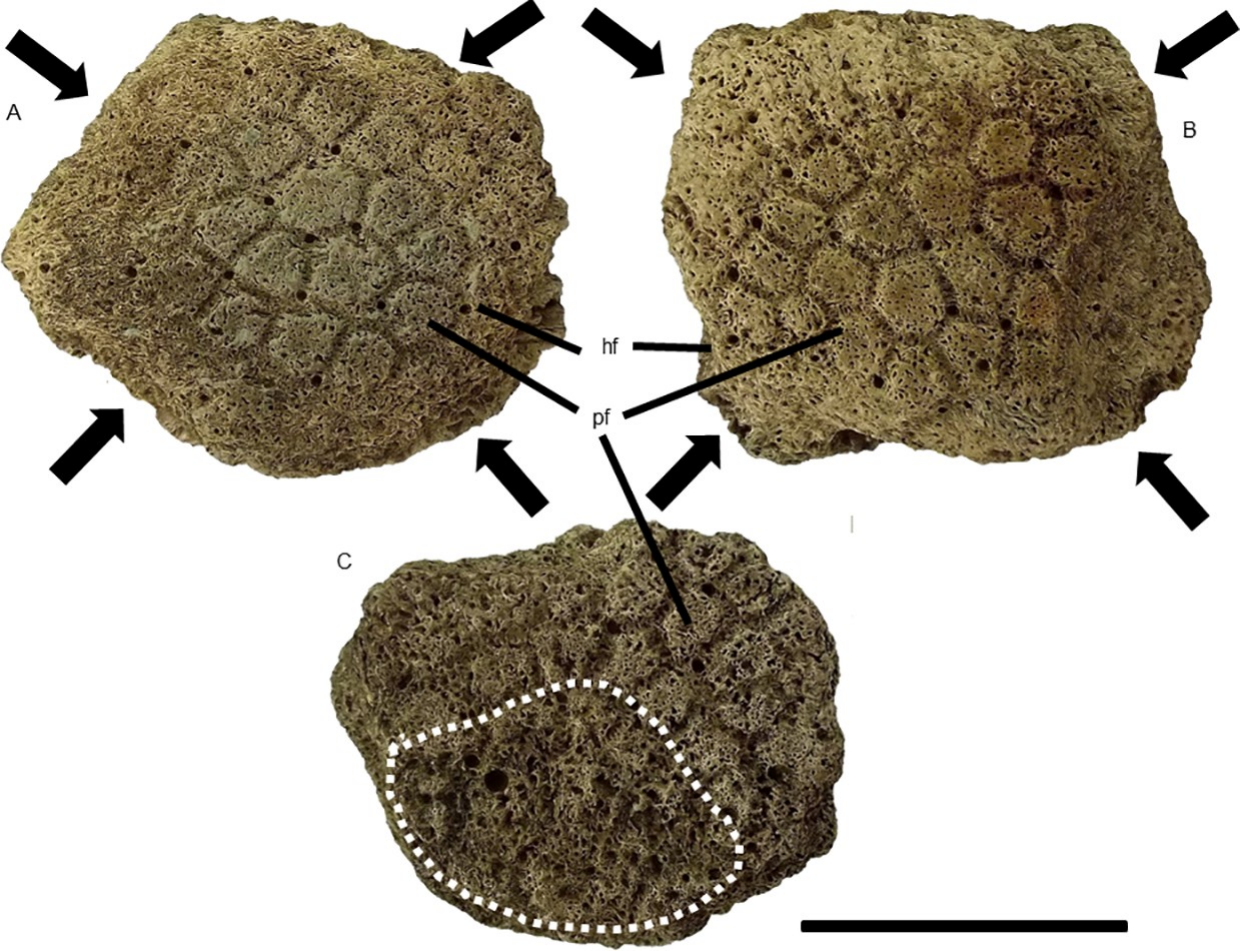
Osteoderms of *Panochthus* sp., external surface. (A) and (B) taphonomic alterations; the arrows indicate the erosion of the ornamentation only in the edges. (C) A pathological condition demonstrated by the irregular and unpolished aspect of the surface that shows spongy tissue highlighted by the dashed line. hf: hair follicle; pf: peripheral figure. Scale bar = 4 cm.

Regarding several osteoderms from Brejo da Madre de Deus, we noted abrasion affecting only the edges, causing an exposition of trabecular bone with a polished aspect (Fig 6A and 6B), but these marks were likely produced by taphonomic processes (i.e., *post mortem*). However, we observed pitting and irregular wearing of the external surface and bone response in osteoderms of this locality, configuring *ante-mortem* reactions (Fig 6C).

**Fig 7:**
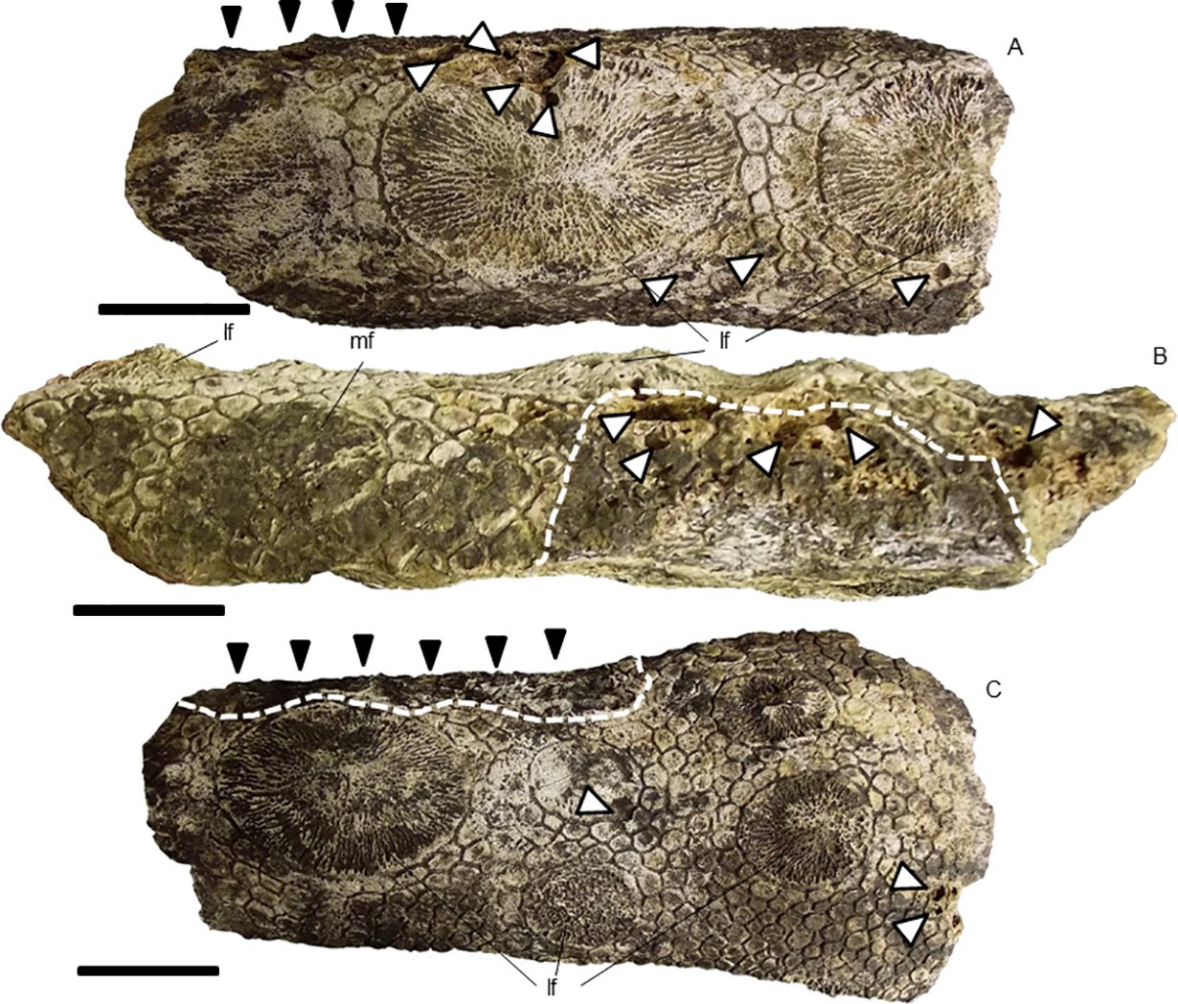
Fragments from lateral region of an uncatalogued caudal tube of *Panochthus* sp. (A) and (C) lateral view, and (B) ventral view; the white arrowheads indicate the cavities generated by fleas; the black arrowheads indicate a depression (see text); the dashed lines delimit the erosion in the ornamentation. mf: main figure, lf: lateral figure. Scale bar = 5 cm.

In the two fragments from the latero-proximal region of the uncatalogued caudal tube of *Panochthus* sp. (Fig 7), only the main and lateral figures from the dorsal region and the main, peripheral and lateral figures from the ventral region were preserved. The primary alteration found in these fragments was the formation of cavities around the lateral figures and in some adjacent peripheral figures (Fig 7A–7C). The alterations were distributed on both lateral regions of the tube, not necessarily affecting the lateral figures. We observed severe damage in the distolateral left region, with the main figure showing a large and deep cavity, measuring approximately two centimeters wide, with microcavities inside that reached the trabecular bone (Fig 7A). Additionally, we noted additional smaller cavities, with trabecular bone exposition and another isolated cavities in the same region, but in a more ventral portion (Fig 7B). However, these cavities did not appear to be generated by horizontal erosion and, therefore were not considered pittings. The other fragment from the lateral region had a depression, associated with erosion marks over the ornamentation, as seen in the lateral region (Fig 7A and 7B).

### *Glyptotherium* sp

We analyzed 1436 osteoderms assignable to *Glyptotherium* sp. with 155 showing alterations: MCC 264-V; MCC 269-V; MCC 271-V; MCC 272-V; MCC 278-V; MCC 279-V; MCC 289-V; MCC 291-V; MCC 293-V; MCC 296-V; MCC 299-V; MCC 300-V; MCC 301-V; MCC 304-V; MCC 306-V; MCC 307-V; MCC 311-V; MCC 501-V; MCC 507-V; MCC 606-V; MCC 612-V; MCC 615-V; MCC 618-V; MCC 622-V; MCC 623-V; MCC 631-V; MCC 673-V; MCC 680-V; MCC 687-V; MCC 688-V; MCC 709-V; MCC 712-V; MCC 726-V; MCC 733-V; MCC 736-V; MCC 741-V; MCC 872-V; MCC 873-V; MCC 1198-V; MCC 1199-V; MCC 1201-V; MCC 1202-V; MCC 1332-V; MCC 1337-V; MCC 1941-V; MCC 1944-V; MCC 1885-V; MCC 1888-V, MCC 1898-V; MCC 1913-V; MCC 1920-V; MCC 1223-V; MCC 1927-V; MCC 1950-V; MCC 1951-V; MCC 1980-V; MCC 1991-V; MCC 1197-V; MCC 1998-V; MCC 2012-V; MCC 2035-V; MCC 2043-V; MCC 2057-V; MCC 2069-V; MCC 2073-V; MCC 2074-V; MCC 2085-V; MCC 2098-V; MCC 2115-V; MCC 2116-V; MCC 2123-V; MCC 2125-V; MCC 2139-V; MCC 2148-V; MCC 2151-V; MCC 2154-V; MCC–2158-V; MCC 2161-V; MCC 2166-V; MCC 2177-V; MCC 2178-V; MCC 2185-V; MCC 2187-V; MCC 2188-V; MCC 2192-V; MCC 2196-V; MCC 2206-V; MCC 2208-V; MCC 2213-V; MCC 2229-V; MCC 2225-V; MCC 2236-V; MCC 2375-V.

The carapace osteoderms of the genus *Glyptotherium* presented the typical rosette ornamentation pattern on their external surface, including: a centrally positioned main figure delimited by a central sulcus, six to eight peripheral figures separated by the radial sulci, and hair follicle pits placed in the intersection of the central and radial sulci. Field records report that these osteoderms were collected in association with endoskeleton elements, which included various paired elements showing anatomic and ontogenetic correlation (e.g., left and right tibia-fibulae) and might belong to a single individual [24]. Different types of arthritis were identified in several bones of this presumed individual [24].

#### Description of the lesions

**Fig 8:**
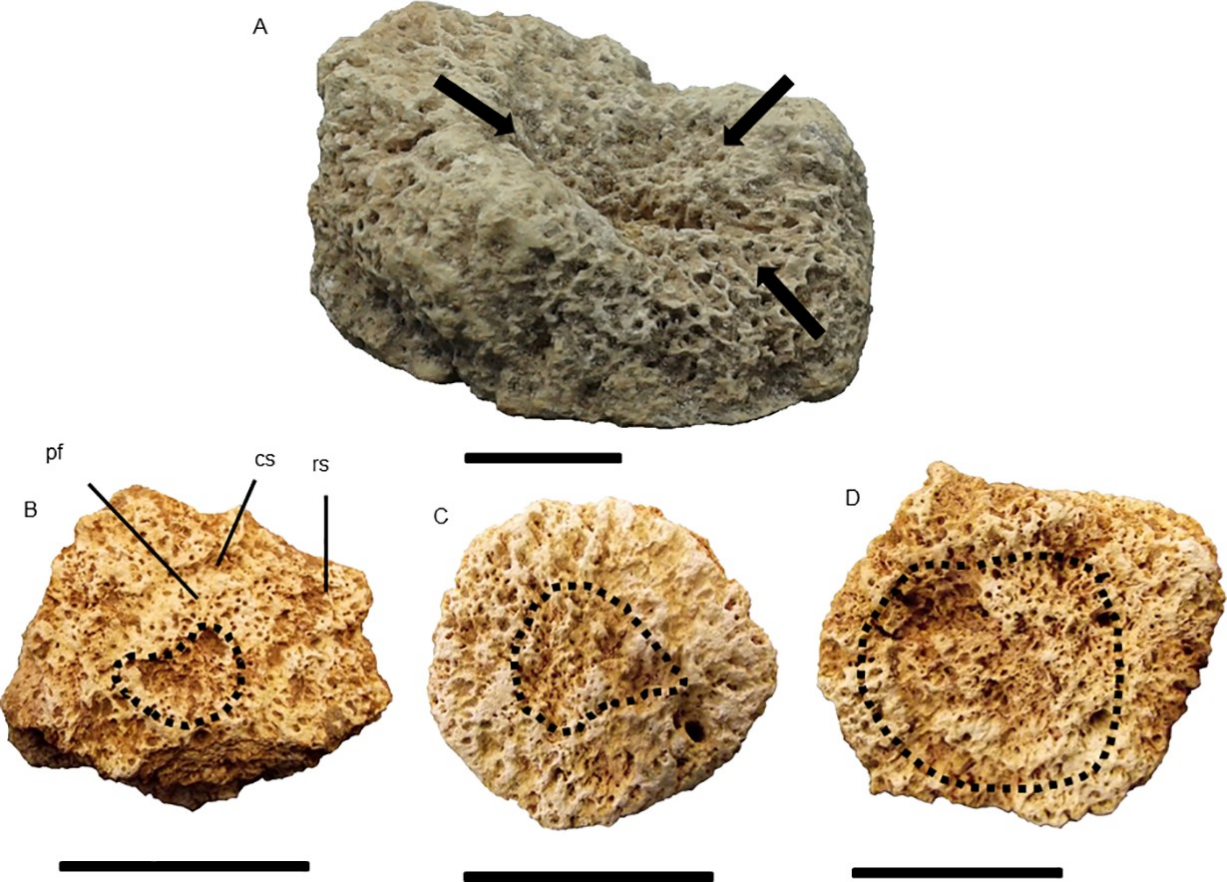
Osteoderms of *Glyptotherium* sp., external surface. **(**A) MCC 2221-V, severe pitting process (arrows). Stages of the pitting process indicated by dashed line in B (initial, MCC 668-V), C (intermediary, MCC 631-V) and D (advanced, MCC 2229-V). Scale bars: A = 3 cm; B = 4 cm.

**Fig 9:**
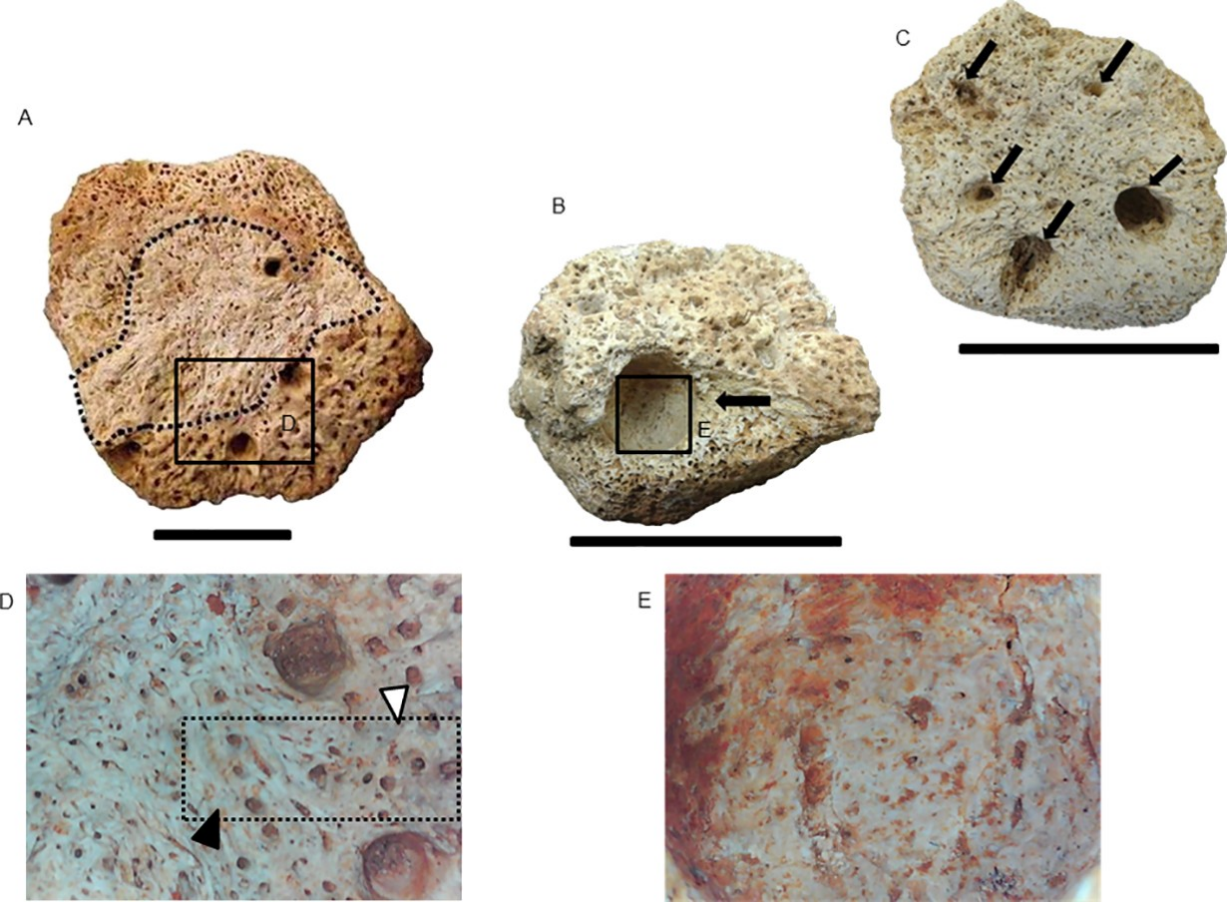
Osteoderms of *Glyptotherium* sp, external surface. (A) MCC 2375-V, bone response area (dashed lines); note the level of reshuffle created by calcium deposition. (B) and (C) perforations produced by fleas in MCC 2565-V and MCC 1198-V (arrows). (D) Digital microscopy image of MCC 2375-V showing the edge (dashed line) between the surface with the bone response (black arrowhead; note the deposition of bony material with a contorted aspect) and the normal surface (white arrowhead) (Zoom: 25x). (E) Interior of the flea perforation in MCC 2565-V (Zoom: 40x). Scale bars: A = 3 cm; B and C = 4 cm.

Pittings of variable sizes were the primary alterations on the osteoderms of this taxon, bearing irregular and/or regular furrows in or outside them. Several osteoderms showed multifocal lesions. Notably, in osteoderms severely affected by pitting, a notable growth of the cavity occurred toward a point at which it became deeper, generating a slope, as observed in MCC 2221-V (Fig 8A). In some cases, the erosion on the surface of osteoderms did not necessarily create a cavity, whereas in others, the erosion produced a large damage that obliterated the surface completely. Some osteoderms showed signs of bone response in the form of calcium deposition in places with severe spongy bone exposition (MCC 2375-V; Fig 9A). In digital microscopical view, we observed the same twisted and contorted pattern of filaments overlapping the spongy bone, as observed in specimen MCC 1653-V (*Panochthus*; Fig 3).The pittings observed in the examined osteoderms were in initial, intermediary and advanced stages, as exemplified by MCC 668-V (Fig 8B), MCC 631-V (Fig 8C) and MCC 2229-V (Fig 8D), respectively, resulting in partial or total loss of the ornamentation. The areas on the external surface of osteoderms with the ornamentation completely obliterated showed spongy bone expositions with varied extensions.

Several osteoderms showed well-delimited circular orifices that penetrate the cortical bone layer reaching the spongy bone. Moreover, the internal surfaces of these orifices had a polished aspect, as observed in MCC 2565-V and MCC 1198-V (Fig 9B and 9C, respectively), some coinciding with the enlargement of hair follicle pits (Fig 9B). We considered that these orifices were not pittings because they did not cause a uniform wearing of the surface, such as a cratering. Another peculiar characteristic was that they were not always associated with loss of the ornamentation in the osteoderms in which they occurred (MCC 1198-V). Digital microscopy images of the perforation in MCC 1198-V showed no bone growth in the perforation or on its edges, but with some furrows and other small cavities inside, and although clearly penetrating the spongy bone area, the perforation showed no exposition of trabeculae in its internal walls (Fig 9E), which had a polished aspect. As in some specimens of *Panochthus* (MCC 1653-V and the posterodorsal region of the carapace), the portion covered by the calcium deposition had a denser aspect than that of the adjacent surfaces (Fig 9D).

### Pachyarmatherium brasiliense

Material analyzed—55 isolated osteoderms of which 10 showed alterations: DEGEO-UFPE 7410; DEGEO-UFPE 7423; DEGEO-UFPE 7424; DEGEO-UFPE 7426; DEGEO-UFPE 7432; DEGEO-UFPE 7264; MCC 1515-V; and three uncatalogued specimens deposited in MCC.

Compared with the glyptodonts previously described, *P*. *brasiliense* had smaller hexagonal osteoderms, some proportionally thicker, with smooth surface, main figure displaced posteriorly and polygonal peripheral figures [13]. We analyzed 33 osteoderms from Fazenda Nova, of which nine presented alterations, and 22 from Lajedo Escada, of which only one showed alterations.

#### Description of the lesions

**Fig 10:**
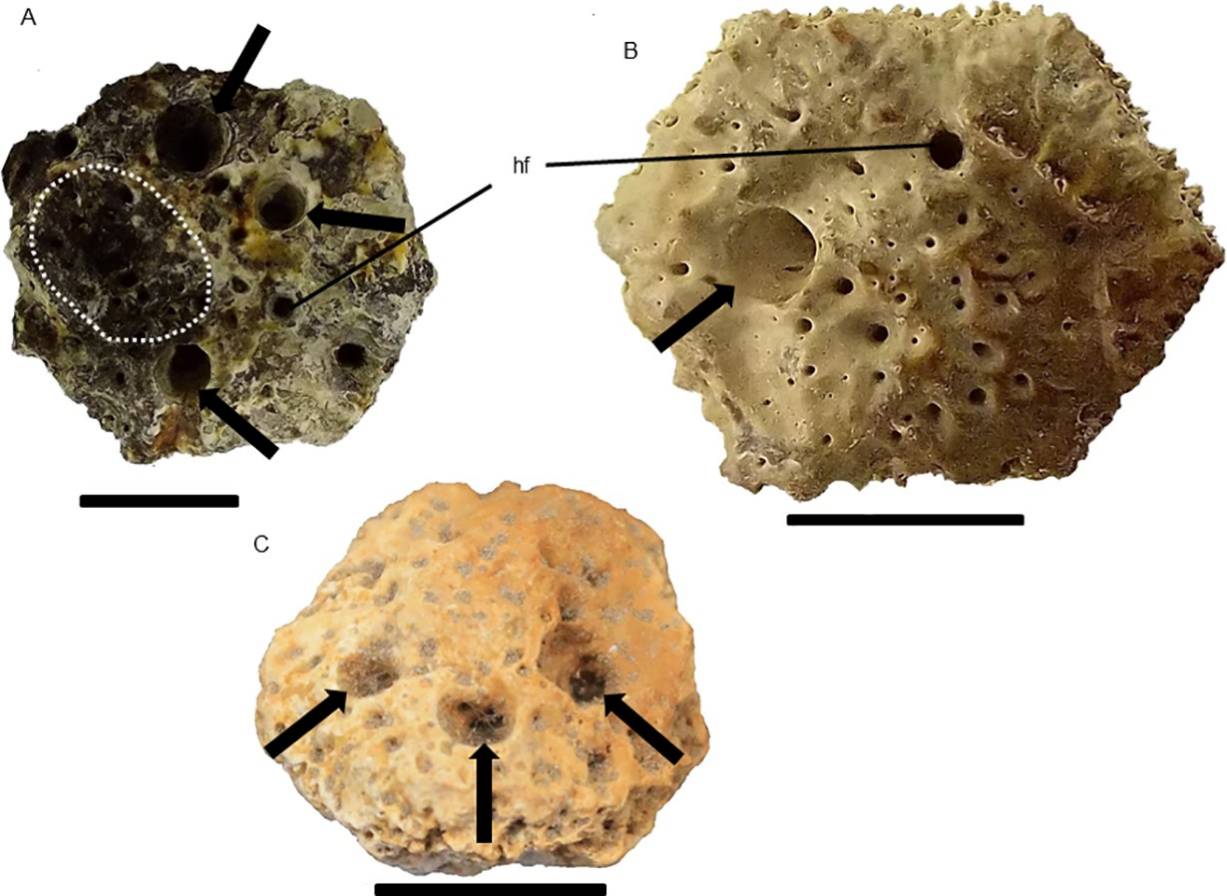
Osteoderms of *P*. *brasiliense*, external surface. The arrows indicate flea perforations in (A) DEGEO-UFPE 7266, (B) DEGEO-UFPE 7410 and (C) MCC 1515-V. In (A) the dashed line delimits the wear in the main figure probably caused by pitting. hf: hair follicle. Scale bar = 1 cm.

The osteoderms from the two localities showed similar alterations to one another and to those of *Glyptotherium* sp. Most alterations corresponded to well-delimited circular cavities on the osteoderm surface, with some, as observed for *Glyptotherium* (see above), representing enlargements of the hair follicle pits. For DEGEO-UFPE 7266, DEGEO-UFPE 7410 and MCC 1515-V (Fig 10), measurements indicated that these enlargements were as large as those observed in *Glyptotherium* (Figs 8 and 9). As in *Glyptotherium*, loss of ornamentation did not necessarily occur in association with the circular perforations. The small erosions on the main figure were a second type of alteration observed, which were not as extensive as in *Glyptotherium*, showed punctual expositions of trabecular bone tissue by excavation, representing probable pittings (Fig 10A).

## Discussion

### Evidences produced by flea infection

In all taxa, we observed marks consistent with a flea infection based on previous works [33, 10, 8]. Fleas are obligatory parasites of many groups of mammals and birds [34]. Among the studied materials, these marks occurred in isolated osteoderms of *Glyptotherium* and *P*. *brasiliense*, and caudal tube fragments of *Panochthus*. They consisted of perforations that penetrated into the bone structure of osteoderms.

In the lateral figures of caudal the tube of *Panochthus*, the flea infection marks were circular cavities, with minor cavities inside of them or in other points of the tube, similar to those figured for late Miocene armadillos (*Chasicotatus ameghinoi* and *Vetelia perforata*), which were attributed to fleas of the genus *Tunga* (See Fig 2E in[8]). This genus includes an extant species with proven ability to produce bioerosion in osteoderms of cingulates (*Tunga perforans*; see [35]). In isolated osteoderms of *Glyptotherium* and *P*. *brasiliense*, we also observed alterations comparable with those described for *C*. *ameghinoi* and *V*. *perforata* [8] and particularly for the extant armadillo *Zaedyus pichiy*, for which infections by fleas of the genus *Tunga* are also reported (See Fig 2 in [9]). In the osteoderms of *Glyptotherium* and *P*. *brasiliense*, these alterations consisted of well-delimited circular perforations, with most representing the enlargement of hair follicle pits.

A comparison among the perforations in exoskeleton elements of *Panochthus* sp., *Glyptotherium* sp. and *P*. *brasiliense* attributed to fleas and similar lesions described in previous work [8] revealed that in all cases, the perforations: (i) were consistently circular; (ii) had the diameter of the external opening broader than that of the internal, forming a tapered orifice; and (iii) were mostly isolated and, in few cases, associated (i.e., with more than one per osteoderm). In some osteoderms of living armadillos the perforations reach the internal surface, that is, the bioerosion crossed the osteoderm thick entirely [33]. By contrast, the perforations observed in the osteoderms analyzed in this study did not reach the internal surface of the specimens; instead, they ended in chambers inside the osteoderms, as in some cases also reported for living [33] and extinct armadillos [8]. This condition might be explained by differences in osteoderm thickness, because both *Glyptotherium* and *P*. *brasiliense* have osteoderms much thicker than those in the dasypodids analyzed in previous studies [33, 10, 8].

In this study, the flea perforations in the osteoderms of *Glyptotherium* and *P*. *brasiliense* ranged from one to five. This condition contrasts with the range registered for the extant armadillo *Z*. *pichiy* (one or two per osteoderm; [10]). At first glance, the size of the osteoderm offered a reasonable explanation for such distinct frequency per osteoderm: the *Glyptotherium* osteoderms have a greater superficial area and are thicker than those of *Z*. *pichiy* and therefore could offer a much larger area for the infesting fleas. The same explanation applies to the osteoderms of *P*. *brasiliense*, which although smaller than those of *Glyptotherium*, are considerably larger than those of armadillos for which similar alterations are described (*C*. *ameghinoi*, *Z*. *pichiy*).

The diameter of the cavity generated by fleas represents additional evidence to reinforce our diagnostic. One study registered diameters ranging from 1.97 to 2.92 mm [10]; similarly, perforations from 1.1 to 3.55 mm were found for *C*. *ameghinoi* and *V*. *perforata* [8]. Regarding the perforations in the osteoderms of *P*. *brasiliense*, we found diameters between 2.1 and 4.3 mm (DEGEO-UFPE 7266 specimen, Fig 10A), which are values partly consistent with those registered by the mentioned authors. For *Glyptotherium*, we found some perforations with diameters similar to those noted above, but we also found some perforations with much greater diameters. For example, in the specimens MCC 2565-V and MCC 1198-V (Fig 9B and 9C), for instance, we measured the greatest diameters among all infected osteoderms, ranging from 8 to 9 mm.

Following the ichnotaxobases classification proposed by [36], the process of bone removal creates a cavity in the formation of the chamber (see Fig 8B). The formation of the chamber by the flea is caused by neosomy in its reproductive cycle. The neosomes are organisms, in this case, fleas, that suffer a radical change during development acquiring a new morphological structure in the metamorphosis process [37]. Initially, the pregnant female penetrates into the host through bone perforation and initiates the neosomy [35]; the chemical or physical bioerosive mechanism by which the flea perforates the bone is unknown. After laying eggs, with the development of the neosome, the flea dies [38].

Traditionally, the identification of *ante-mortem* alterations requires the observation of a bone response [39]; however, no previous authors who registered bioerosion by fleas mentioned evidence of such process. Additionally, the figures in these works [33, 10, 8] do not show any obvious evidences of bone response. Similarly, we did not observe traces of bone response associated specifically with the lesions attributed to fleas. In fact, the living species *T*. *perforans* was described based on its ability of bone perforation [35], although associated bone response was not detected; at the end of the process of neosomy, the appearance of sequels, such as healing or infections, can occur or not [40].

The absence of bone response associated with the perforations on the external surface that were attributed to fleas might cast doubt on the differentiation between *ante-* and *post-mortem* alterations. Therefore, some additional comments on the alterations that we attributed to fleas are necessary. Several works cite *post-mortem* alterations in bone that, such as perforations generated by necrophagic coleopteran larvae in fossil vertebrate bone (See [41, 35, 42]), which at first glance, might be confused with *ante-mortem* marks, such as those attributed to fleas in this study. However, their internal morphology diverges, because necrophagic larvae construct chambers with a diameter twice the diameter of the opening. Moreover, the cavity has an ellipsoidal format (*Cubiculum levis*; see Fig 4 in [43]). By contrast, fleas produce cavities with the diameter equal or minor to the opening with a circular outline, as observed in the material in this study (Fig 9B). A possible reason for this difference is the finality of the excavation. The necrophagic larvae reach for the bone medullary channel to feed on the bone marrow, and consequently, the perforations reach deep portions of the bone and are interconnected (see Fig 2 in [42]). By contrast, fleas are less invasive because the bone is used as a reproduction site and for feeding on blood, and therefore, the cavity created is not required to reach deeper regions of the bone.

Another notable character that discriminates *ante mortem* bioerosion by fleas from *post mortem* bioerosion by necrophagic larvae is the relative number of cavities per osteoderm and their frequency along the carapace. Many cavities in a short area and with small spacing among them would suggest that many larvae actuated in the process. For fleas, few osteoderms show more than one cavity, which may result from competition avoidance behavior. Accordingly, few osteoderms among all analyzed material of *Glyptotherium* and *P*. *brasiliense* had perforations attributable to fleas, which is similar to the conditions previously reported for dasypodids. In fact, a notable detail regarding infection by fleas on dasypodids is the scanty and isolated nature of the perforations produced by these infestations in carapaces of a specimen of the living armadillo *Z*. *pichiy* (see Fig 5 in [9]). Similar lesions are observed in extant armadillos in a recent paper [11], but which does not discuss their distribution over the carapace. However, assessing the figures of the mentioned work (Figs 4G, 5B and 6F in [11]), we note that the lesions have a similar pattern of distribution. Assuming that a similar scanty distribution was also common in fossil cingulates infested with fleas, the low frequency of osteoderms showing this type of perforation in the analyzed material would be explained, with 10 of 55 osteoderms of *P*. *brasiliense* and only nine of 1436 of *Glyptotherium*. Additionally, note that all perforations attributed to fleas in this study occurred only on the external surface and were not observed in the underside or in the lateral regions of osteoderms, which is an unlikely condition had they been caused by necrophagic larvae or other *post mortem* process.

We mentioned previously that the *Glyptotherium* osteoderms in this study were found associated with endoskeleton bones, and these bones were examined with a perspective to identify *ante mortem* alterations [24]. After we reanalyzed these fossils, *post mortem* alterations were not found in the endoskeleton elements assigned to *Glyptotherium* [24] that could be attributed to insect larvae similar to those reported in the works mentioned above. This finding and the morphological differences indicate that such agents did not make the circular perforations on the osteoderms of this genus in this study. Conversely, none of these endoskeleton elements found associated with the osteoderms of *Glyptotherium* and *Pachyarmatherium* in the Lajedo da Escada site had perforations similar to those here attributed to fleas. Some of the elements of the exoskeleton of *Glyptotherium*, in addition to skeletal elements of other Pleistocene taxa from the same site, showed tooth traces likely produced by scavengers and predators [44], but these marks were scratch-like and much different from the perforations and other alterations described here.

Some marks produced by teeth of carnivores in bones bears resemblance to flea perforations, such as those of the ichnospecies *Nihilichnus nihilicus* [45]. For the cavities produced by the genus *Tunga*, the cortical bone is reached and the diameters are similar (2–10 mm). However, *N*. *nihilicus* is more frequent in long bones than in short bones and as far as we know, not yet reported for osteoderms. Morphologically, *N*. *nihilicus* differs from the flea perforations described in this study in having irregular edges and in some cases, associated microfractures (see Fig 6 in [45]). Lastly, the external morphology found in some marks of *N*. *nihilicus* is triangular or half-circular, which clearly differs from the well-delimited circular chambers left by the fleas.

Lesions in the carapace of a living armadillo similar to those discussed above were described in an early work [46] and attributed to mites. These marks consisted in the widening of the diameter of hair follicle pits with loss of ornamentation around some enlarged orifices (see Figs 29b, 33 and 36 in [46]). This same work reported similar alterations in a glyptodont (*Glyptodon*) and inferred that mites also produced these alterations. In fact, mites are among the arthropod parasites living on armadillos, but they do not perforate their carapace [8]. Therefore, we believe that these reported alterations are compatible, in terms of morphology and mode of occurrence, with those described in this study and therefore likely represent an additional case of flea infection in glyptodonts.

Considering the enlargement of the hair follicle pits, these pits were likely a preferred flea entrance place in the osteoderms of large extinct cingulates such as in living cingulates. This enlargement was most like caused by neosomy, although the flea entrance was not exclusively through the hair follicles, because not all cavities observed coincided with these structures; in some cases, the cavity produced by fleas was on the main figure (Fig 9B). In all cases, the perforations produced by fleas were three to fourfold wider than the normal hair follicle pits and were not confined to inside the main sulci, reaching portions of the peripheral and central figures, in contrast to the normal hair follicle pits (see Fig 10A and 10B).

The Tungidae family encompasses fleas that infect cingulates and is the only group that shows neosomy except for some members of the *Neotunga* genus (Pulicidae), which are restricted parasites of pangolins and phylogenetically distant of Tungidae [34]. Four of the 13 species in the genus *Tunga* infect living cingulates (see [38], Table 4), with *T*. *penetrans* the most common. Until now, flea infections were described only in small dasypodids (extinct and extant); thus, the fossils described here represent the first case of *Tunga* in glyptodontids and in a non-glyptodontid cingulate with large size (*Pachyarmatherium*).

### Etiology of the pittings

In *Panochthus*, we noted the formation of pitting in the main figure of isolated osteoderms and on the carapace (MCC 1603-V), with the pitting all located in the osteoderms of the lateral regions (Fig 4), as the primary alteration in these bones. In *Glyptotherium*, we observed initial (Fig 8B), intermediary (Fig 8C) and advanced (Fig 8D) stages of pitting, with spongy bone exposition in all cases. The pittings and the irregular obliteration of the ornamentation associated with the former were not attributable to fleas, although fleas could have fundamental participation in their origin acting as vectors of opportunistic micropathogens that cause dermal lesions, such as *Staphylococcus aureus* and *Clostridium tetani* [38]. Additionally, fleas transmit the fungus *Paracoccidioides brasiliensis*, responsible for some dermatitis and the bacteria *Clostridium perfrigens* [47]. The erosions of the ornamentation observed in some of the specimens analyzed (MCC 668-V, MCC 631-V and MCC 2229-V) were similar to those attributed to bacterial attack, particularly by *Mycobactherium leprae*, although leprosy does not cause significant alterations in cingulates [48]. A case of sporotrichosis in living armadillos was reported that destroyed a wide extension of epidermis by the opportunist fungus *Sphorothrix scheenckii*, which is very common in osteological infections, generating superficial erosion [49]. However, because of the lack of diagnosable features for such infections in osteoderms analyzed in this study, we could not conclude what type of pathogen was responsible for the pitting observed in the fossils.

The cave environment of Lajedo da Escada in which the osteoderms of *Glyptotherium* sp. and some specimens assigned to *P*. *brasiliense* were found requires considering the possibility that some alterations observed on these osteoderms were caused by *post mortem* conditions in such environments. Note that inside limestones basements, the formation of the caves, carbonic acid (H_2_CO_3_) mediates the formation of the caves by slowly corroding and perforating the limestones rocks [50]. In fact, we noted that the osteoderms of *Glyptotherium* sp. showed traces of corrosion by acid, such as powder release, as in a chalk.

In this context, we could not discard that carbonic acid contributed to the loss of ornamentation associated with pitting in *Glyptotherium* sp. osteoderms, although the effect of acid was not primarily responsible. As we noted earlier, some osteoderms collected in Lajedo da Escada showed bone response, which is clear evidence of an *ante mortem* process (Fig 9A). In these cases, the acid likely magnified the marks left by the pathological process, that is, the injury appeared first, in life, and then, after death, the acid enlarged the diameter of the cavities by corrosion. The fact, mentioned above, that only their external surfaces were affected is important evidence showing that *post mortem* corrosion by acid was not the primary explanation for the alterations observed in the osteoderms of *Glyptotherium* sp. from Lajedo da Escada. Although acids can mimic bone infections and create pitted surfaces [51], in such cases, we would expect that all surfaces of the affected osteoderms would show evidence of corrosion, which was not observed on the internal surfaces in the studied specimens.

We must emphasize that the irregular obliterations of the ornamentation along the carapace and some fragments of *Panochthus* sp. could not be considered pittings, although they were associated with them. These alterations had a spread extension; whereas pitting is a punctuated erosion of bone surface. Another difference was the formation of crests associated with a wide erosion of the ornamentation and holes for pitting.

### Paleoecological and evolutionary implications

We recorded new potential cases of disharmonic interspecific interaction (parasitism) between cingulates and fleas for taxa previously not reported for this type of alteration. Fleas of the genus *Tunga* might have been biogeographically widely distributed and a common parasite of most or all cingulate main linages during the evolution of this group, at least since the late Miocene [8]. All examined taxa (*Panochthus* sp., *Glyptotherium* sp. and *P*. *brasiliense*) showed perforations attributable to neosomy (flea egg-laying), similarly to the cases involving other extinct (*C*. *ameghinoi* and *V*. *perforata*) and extant (*Chaetophractus villosus)* species [8, 10]. Moreover, this work expands the distribution area of species of the genus *Tunga* able to perforate bones, which was restricted to Argentina [10, 11, 8], reporting their occurrence also in environments of the Brazilian Intertropical Region.

We can envisage the success of Tungidae fleas when we correlate flea temporal distribution along the Cenozoic [52] and its singular ability to infect the hosts. The biochron ranges from late Miocene [8] to Holocene [10, 11], including the new cases from the Pleistocene described here. This ability to perforate bones could have conferred advantages over other fleas that might have been limited to the dermic surface. This adaptation might have avoided competition for ecological niches, increasing availability of habitat and resources for reproduction.

As stated previously, among the known species of the genus Tunga, only *T*. *perforans* shows the bone perforation ability. However, we cannot discard the existence of other species within Tungidae with the same capacity because of the considerable time scale involved in the known interaction between this family and cingulates, whose most ancient record dates from the late Miocene [8]. If we consider *T*. *perforans* as the only species infecting cingulates since that period, we must assume a wide evolutionary stasis for a species with a fast life cycle and expansive geographic distribution, which is an unlikely scenario. In short, we consider the hypothesis that the bone perforation ability of Tungidae fleas persisted during this entire time interval but was not necessarily restricted to *T*. *perforans*.

## Conclusions

We report on ectoparasitism and infections in exoskeleton elements of the large-sized *incertae sedis* cingulate *P*. *brasiliense* and the glyptodonts *Panochthus* sp. and *Glyptotherium* sp. For these taxa, we described probable cases of alterations caused by fleas and by infection of bacteria or fungi, sometimes in the same element. We described the first case of infection by fleas of the genus *Tunga* in *Glyptotherium* sp., *Panochthus* sp. and *P*. *brasiliense*, which until now, was reported only for small sized cingulates (extinct and extant). We also report pittings in osteoderms of *Glyptotherium* sp., *Panochthus* sp. and *P*. *brasiliense* that might be attributable to bacterial or fungal infections, which were previously reported only in osteoderms of ankylosaurid dinosaurs. These fungal and bacterial infections might be related to the presence of fleas in some cases, but the evidence was not available to allow a more decisive conclusion on the possibility of this interaction.

These new occurrences revealed a wider geographical distribution for the fleas of the genus *Tunga* during the Cenozoic, because previous reports (in extant and extinct cingulates) were confined to more southern areas. Additionally, the cases of flea infestation reported here, together with previous ones, represent an interesting case of coevolution between Tungidae and cingulates.
